# Supplementary material for: Signatures of the Kondo effect in VSe2
Source: Sci Rep. 2017 Sep 8;7:10964. doi: 10.1038/s41598-017-11247-4 (PMC5591204; doi:10.1038/s41598-017-11247-4)
Supplement: Supplementary file 1 — Supplementary Information [file 41598_2017_11247_MOESM1_ESM.doc]

Supplementary Information: Signatures of the Kondo effect in VSe_2_

**Sourabh Barua^1,*^, M. Ciomaga Hatnean^1^, M. R. Lees^1^ and G. Balakrishnan^1,*^**

^1^Department of Physics, University of Warwick, Coventry, CV4 7AL, United Kingdom

^*^S.Barua.1@warwick.ac.uk, G.Balakrishnan@warwick.ac.uk

**Resistivity versus temperature for additional samples**

The temperature dependence of the resistivity of several crystals from two different growth batches, Batch 1 and 2, were measured for this study. Here, we present the ρ(*T*) data for two samples taken from each batch. All the samples show an upturn in the resistivity at low temperatures. The low temperature resistivity for these samples and the fits to the data using the Hamann equation (Equation 3) and the modified Hamann equation (Equation 3 with *T* replaced with *T*_eff_) are presented in Figs S1-S4.

As described in the main text, the Hamann fit is performed by first determining the electron-phonon contribution to ρ(*T*) by fitting the resistivity between 10 and 20 K using an expression of the form *a* + *bT*^n^. The values of *b* and *n* are then used for fitting Equation 3, which includes the Hamann equation and a term describing the electron-phonon scattering. The parameters *T*_K_, and *S* obtained from the Hamann fit are then used in fitting the modified Hamann equation. The Hamann fit is performed over the temperature range 2 to 10 K and the modified Hamann fit is carried out over the temperature range 0.5 to 10 K.

Figure S1 shows the temperature dependence of the resistivity, along with the fit to the Hamann equation, for a Sample 1B from Batch 1. (Sample 1B comes from the same batch as the sample whose resistivity versus temperature is shown in Fig. 3 of the main text). The resistivity of Sample 1B was measured down to 2 K and only a Hamman fit is performed for this sample as the resistivity only flattens off at lower temperatures. Figure S2 shows the upturn in ρ(*T*) of another crystal (Sample 1C) from Batch 1. The resistivity of this crystal was measured down to 0.5 K and was fitted using both the Hamann and modified Hamann equation.

Figure S3 shows the low temperature upturn in resistivity for a crystal from Batch 2 (Sample 2A). The resistivity of this sample was measured down to 2 K and only a Hamman fit is performed for this sample as the resistivity only flattens off at lower temperatures. Figure S4 shows the low temperature resistivity for a second crystal from Batch 2 (Sample 2B). The resistivity was measured down to 0.5 K and was fitted to both the Hamann equation and the modified Hamann equation. In this sample, the resistivity starts falling once again after it has flattened off at very low temperature. Such behaviour has been noted in ZnMn alloys [Ref. 31 (main text)] due to the RKKY interaction between the magnetic impurities.

The results of the fits to the Hamann equation for the different samples are given in Table S1 and for the fits using the modified Hamann equation in Table S2. The parameters obtained from the fits for the different samples, including the Kondo temperature *T*_K_ and spin *S*, are in reasonable agreement for the different samples.


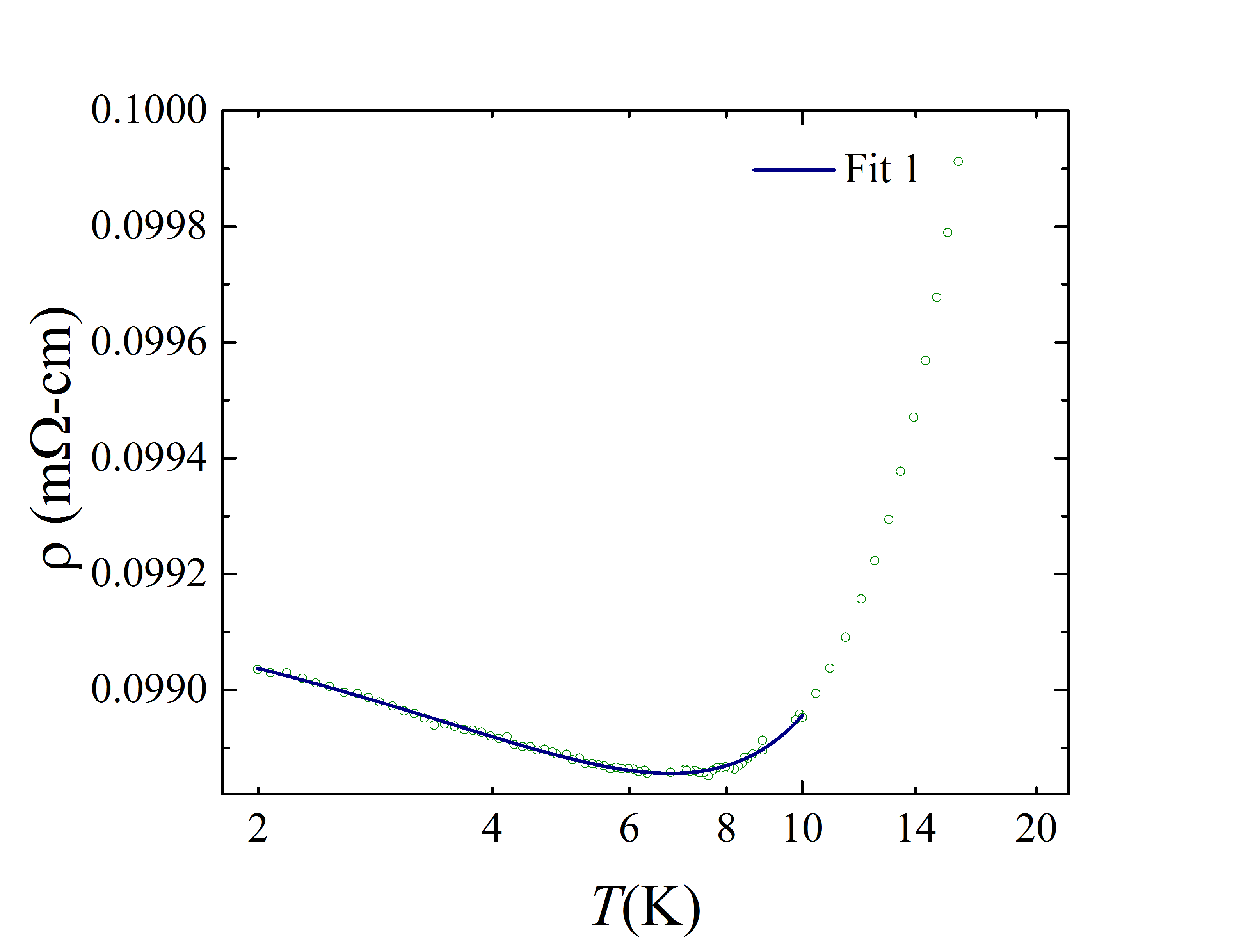


Supplementary Figure S1: Resistivity versus temperature of Sample 1B. The low temperature upturn in resistivity is fitted to the Hamann equation (Fit 1).**
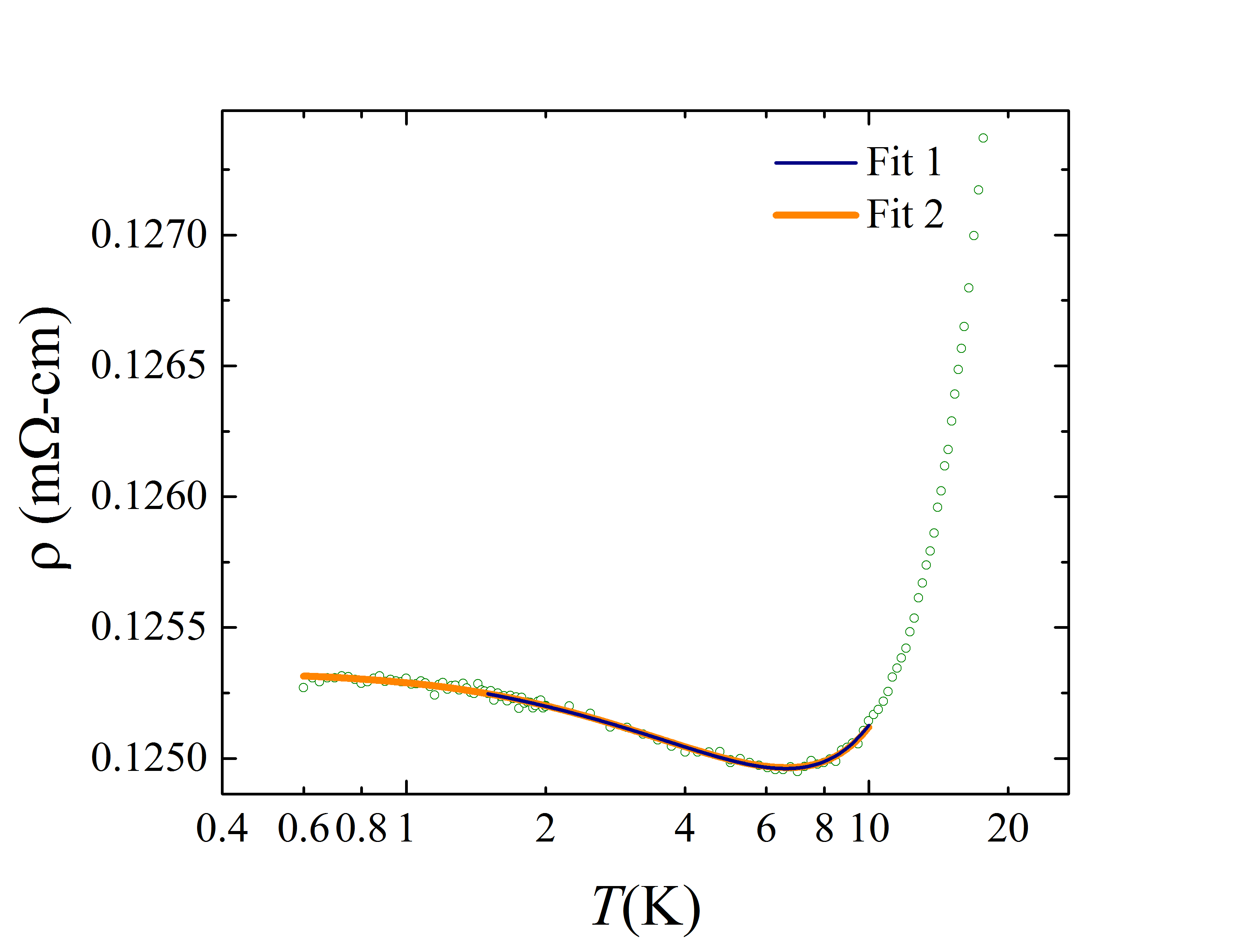
**

Supplementary Figure S2: Resistivity versus temperature of Sample 1C. Fit 1 and Fit 2 use the Hamann and modified Hamann equation respectively.


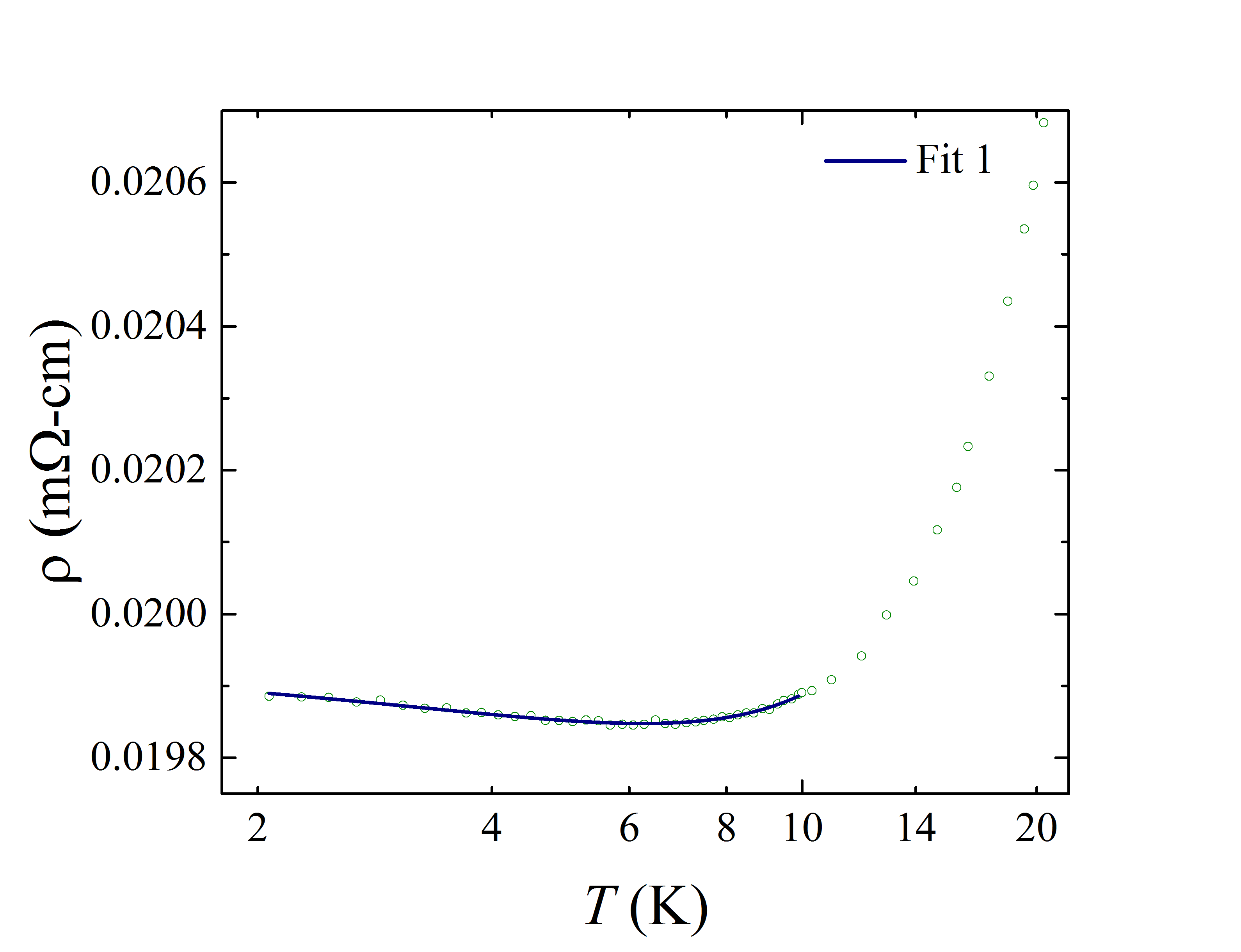


Supplementary Figure S3: Resistivity versus temperature of Sample 2A. The low temperature upturn in resistivity is fitted to the Hamann equation (Fit 1).
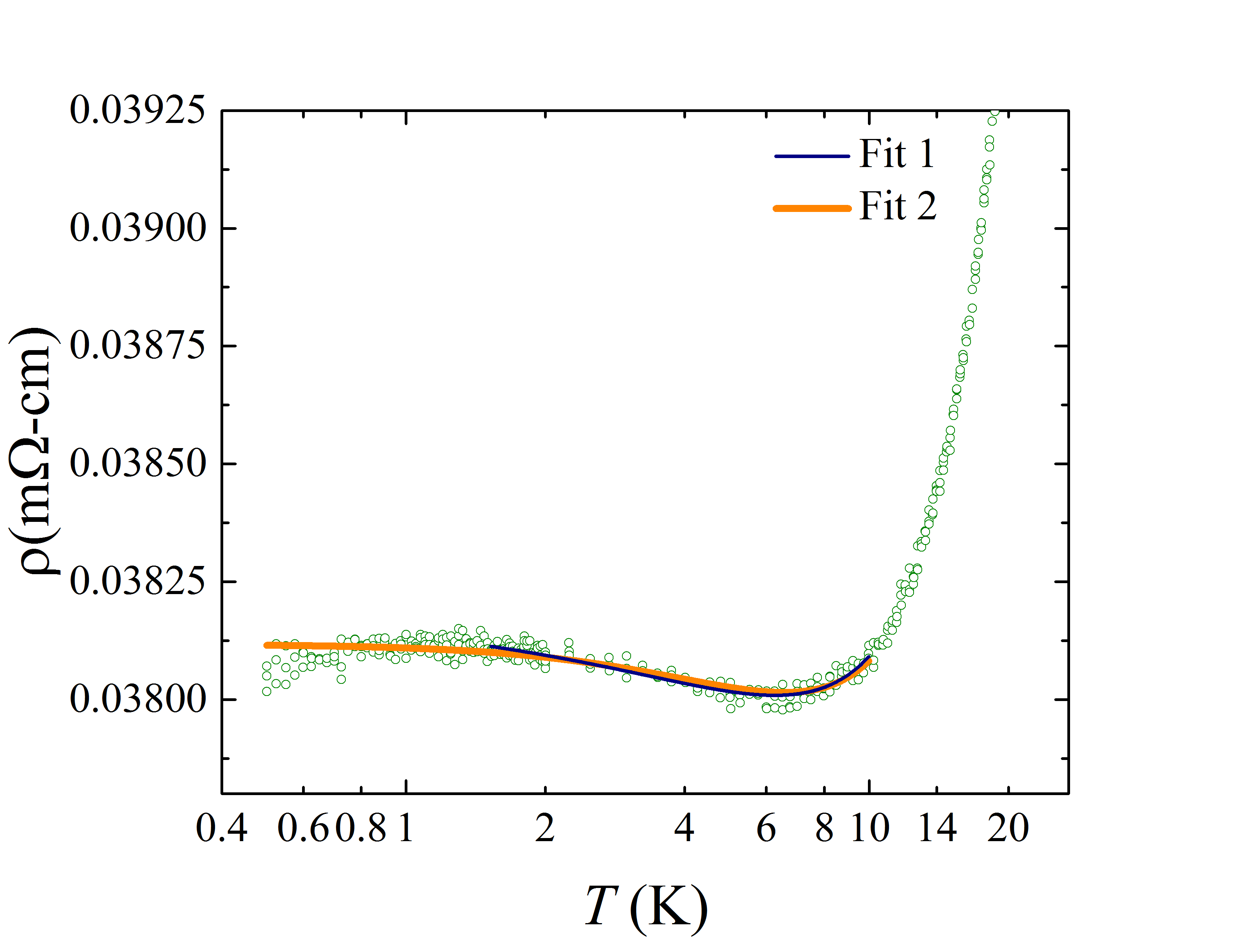


Supplementary Figure S4: Resistivity versus temperature of Sample 2B. Fit 1 and Fit 2 use the Hamann and modified Hamann equation respectively.

Supplementary Table S1: Parameters obtained from the fit to ρ(*T*) using Equation 3 which includes the Hamann equation along with a term that takes into account the electron-phonon scattering.

| Sample | ρ_0_ (Ω-cm) | ρ_b_ (Ω-cm) | *T*_K_ (K) | *S* |
| --- | --- | --- | --- | --- |
| 1B | (1.1 ± 0.2)×10^-6^ | (9.82±0.01)×10^-5^ | 8±1 | 0.3±0.1 |
| 1C | (1.2 ± 0.2)×10^-6^ | (1.243 ±0.002)×10^-4^ | 6±1 | 0.2±0.1 |
| 2A | (2.45 ± 0.04)×10^-7^ | (1.9708 ± 0.0007)×10^-5^ | 6 ± 1 | 0.3 ± 0.1 |
| 2B | (5.3±0.3)×10^-7^ | (3.768± 0.003)×10^-5^ | 7 ± 1 | 0.3 ± 0.1 |

Supplementary Table S2: Parameters obtained from the fit to ρ(*T*) using Equation 3 with the temperature *T* replaced by effective temperature *T*_eff_. This equation is the modified Hamann equation along with a term describing the electron-phonon scattering.

| Sample | ρ_0_ (Ω-cm) | ρ_b_ (Ω-cm) | *T*_W_ (K) |
| --- | --- | --- | --- |
| 1C | (1.31 ± 0.03)×10^-6^ | (1.2423 ± 0.0001)×10^-4^ | 2.0±0.1 |
| 2B | (7.9±0.7)×10^-7^ | (3.758±0.003) ×10^-5^ | 3.5±0.4 |
